# Impact of the COVID-19 pandemic on the severity and management of acute appendicitis

**DOI:** 10.3389/fsurg.2022.981885

**Published:** 2022-09-01

**Authors:** Marion Poget, Roland Chautems, Rémy Kohler, Michele Diana, Alend Saadi

**Affiliations:** ^1^ Surgery Department, Neuchâtel Hospital, Neuchâtel, Switzerland; ^2^ Faculty of Medicine, Geneva University, Geneva, Switzerland; ^3^ Surgery Department, Geneva University Hospitals, Geneva, Switzerland; ^4^ IRCAD, Research Institute Against Cancer of the Digestive System, Strasbourg, France; ^5^ Surgery Department, University Hospital of Strasbourg, Strasbourg, France; ^6^ Obesity and Metabolic Diseases Center, Neuchâtel Hospital, Neuchâtel, Switzerland; ^7^ Faculty of Biology and Medicine, University of Lausanne, Lausanne, Switzerland

**Keywords:** appendicitis, severity, appendectomy, COVID-19, SARS-CoV-2

## Abstract

**Background:**

The literature seems to indicate that the number of appendectomies dropped at the beginning of the coronavirus disease in 2019 (COVID-19 pandemic), while the number of complicated appendicitis increased due to late presentation. In addition, a longer delay before surgical treatment resulted in a higher morbidity. This study aims to compare the number of appendectomies, the severity, and the management of acute appendicitis during the first two pandemic peaks of COVID-19 with those observed during the same seasonal periods in the previous 2 years in a regional hospital in Switzerland.

**Methods:**

We retrospectively reviewed and compared the number of appendectomies, rate of complicated appendicitis, delay to consultation and to surgery, distribution of appendectomies over a 24-h schedule, postoperative outcomes, and rates of overall complications in 177 patients, that is, 66 during the COVID-19 pandemic and 111 before the pandemic.

**Results:**

No statistical difference was found in the number of appendectomies, duration of symptoms before consultation, median time to surgery, number of appendectomies performed outside the usual scheduled time for non-urgent surgery, length of postoperative stay, or the rates of overall complications. However, there was a trend in the rate of complicated appendicitis (*p* = .05).

**Conclusion:**

In spite of a high incidence rate of COVID-19 in our canton, the impact of COVID-19 on our population did not follow the pattern observed elsewhere. The reasons for this might be that people would still present to the emergency department due to less strict social distancing measures. Great availability of emergency operating room may also account for the unchanged delay preceding surgical treatment and complication rates.

## Introduction

The coronavirus disease of 2019 (COVID-19), characterized as a pandemic on 11 March 2020 by the World Health Organization (1), resulted in a worldwide health crisis. Strict sanitary measures implemented by the Federal Office of Public Health allowed to slow the initial progression of Coronavirus in Switzerland (2).

Nonetheless, the Swiss population faced two peaks of COVID-19 infection from March to May and from October to December 2020, leading to an increased number of hospitalizations and mortality (3). It should be pointed out that the canton of Neuchâtel, where our institution is located, was one of the most severely affected cantons in Switzerland, with an extrapolation of COVID-19 cases of 6,692 per 100,000 residents in 2020 (4), hence exceeding the Swiss mean of 5,443 cases per 100,000 residents (5).

As elsewhere, the Hospital of Neuchâtel, a regional, non-university hospital, called off non-emergent surgical procedures and dedicated operating rooms to care for COVID-19 intubated patients, to cope with critical care bed capacity issues (6–8).

Non-urgent surgeries are usually performed from 8 a.m. to 10 p.m. in our institution. During the pandemic, the hospital implemented a non-stop access to the emergency operating room to allow a reduction in the total length of stay for these surgical patients and prevent potential complications caused by a longer delay before treatment.

The hospital is provided with 370 acute care beds, 62 of them being allocated to the service of general surgery, i.e., digestive, thoracic, and vascular. Two wards of surgery were temporarily closed during the first pandemic peak, reducing the number of beds down to 50 for the general surgery, orthopedic, urology, and ear nose and throat services altogether.

A review of the literature at the time of the initiation of our study reported that the number of presentations to the emergency department and the number of appendectomies decreased during the first pandemic peak at the beginning of the year 2020, while the interval before surgical treatment, the rates of perforated appendicitis associated with a late presentation, and the rates of postoperative complications increased (4, 6–15). Similar results have been found in Switzerland: Passoni et al. have reported an increase in complicated appendicitis due to late presentation from April to October 2020, between the two pandemic peaks (16). Texeira Farinha et al. have shown the same between March and April 2020 (17).

A recent study emphasized on the management of acute appendicitis during the pandemic in April 2020 including 66 countries over the world (18). To date, the level of evidence of management of this condition during the pandemic is low, with many recommendations being based on expert opinion and case series.

In the above study, there was an increase in practice of non-operative management with antibiotics in patients with both uncomplicated and complicated appendicitis. This strategy was adopted to reduce resource consumption and avoid unnecessary surgery. Moreover, there was a significant increase in the practice of straightforward open appendectomy for both uncomplicated and complicated appendicitis, thus limiting the potential risk of viral transmission through pneumoperitoneum (smoke and aerosolization).

Screening patients for SARS-CoV-2 preoperatively varied among countries during the early pandemic in 2020 (18, 19): overall, half of surgeons tested symptomatic patients or patients with a suspicion of infection. However, 58.3% respondents from Africa and 27.6% from Latin American did not perform a screening test before surgery.

In our institution, all patients were routinely screened for SARS-CoV-2 with PCR alone preoperatively.

This study aims to evaluate the impact of the first two COVID-19 pandemic peaks on the number of appendectomies, the severity of acute appendicitis, the delay to presentation and surgical treatment, and the postoperative outcomes, as compared with the same seasonal periods during the preceding two years.

## Materials and methods

Demographic and disease-related data of 66 patients who underwent emergency appendectomy at the Hospital of Neuchâtel were retrospectively collected from March to May 2020 and from October to December 2020, during the first two pandemic peaks of COVID-19 (COVID cohort). The inclusion criteria were adult and pediatric patients over 13 years of age, operated upon for acute appendicitis at the Hospital of Neuchâtel with a diagnosis of appendicitis confirmed by the pathology report. Non-inclusion criteria were tumors, endometriosis of appendix, and the absence of a signed consent form.

Data were compared with those of 111 patients in 2018 and 2019 over the same periods (pre-COVID cohort), as a historical comparison. All consecutive patients were included in the study.

The primary endpoint was the number of appendectomies. Secondary endpoints included the presence of a complicated appendicitis (perforation, abscess, and phlegmon) confirmed during surgical intervention, the delay to emergency department presentation, the delay from admission to surgery, the number of appendectomies performed outside the usual scheduled time for non-urgent surgery, the length of postoperative stay, and the postoperative complications.

In our institution, follow-up is achieved either by the surgeon or by Swissnoso (20), the national center for infection prevention, upon a phone call when patients cannot meet the surgeon 1 month after surgery. Whenever a postoperative complication was suspected, the patients were referred to the hospital. Hence, 93.9% of patients were being followed up for at least 1 month postoperatively between 2020 and 2021 and 93.8% between 2011 and 2021.

Categorical data were quantified as counts and percentages and compared with the use of the Chi-square test. Continuous data were displayed as means with standard deviations or medians with interquartile range according to their normal distribution or otherwise and compared using Student’s *t*-test or non-parametric tests, as appropriate. Statistical analyses were performed by using Prism version 8.4.3 (GraphPad Software, San Diego, CA, USA).

All statistical tests were two-sided and a *p*-value <.05 was considered statistically significant.

This study was reviewed and approved by the local Commission on Ethics in Human Research (CER-VD, number 2021-01554).

Informed consent was obtained from all individual participants in this study.

## Results

**Figure 1:**
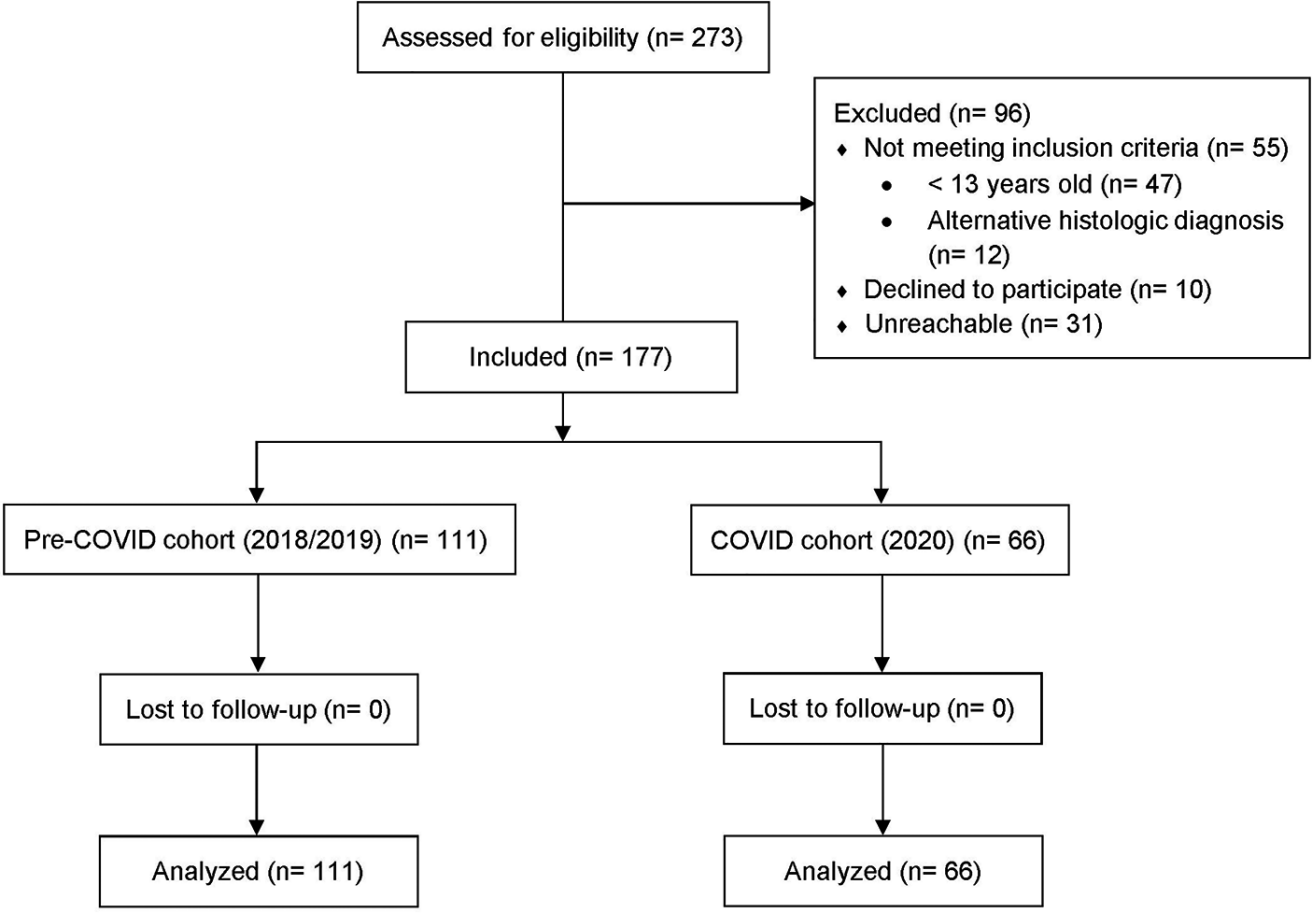
Patient flow diagram.

**Figure 2:**
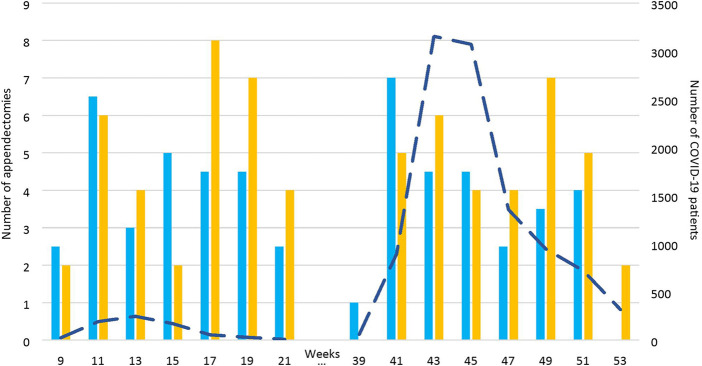
Number of appendectomies in 2018/2019 and 2020 (columns) as compared with the number of COVID-19 patients in 2020 (dotted line).

One hundred and seventy-seven patients were included, i.e., 66 in the COVID cohort in 2020 and 111 in the pre-COVID cohort (58 in 2018 and 53 in 2019). A patient flow diagram is represented in Figure 1. Figure 2 displays the mean number of appendectomies in 2018 and 2019 and the number of appendectomies in 2020 along with the number of COVID-19 patients during the two peaks in 2020. No statistically significant association was found between the number of appendectomies and the COVID-19 peaks. The demographic and clinical data of the patients are described in Table 1.

**Table 1:** Demographics, severity of appendicitis, delay before consultation, delay to surgery, and postoperative outcomes in pre-COVID (2018/2019) and COVID (2020) cohorts.

	2018/2019 (*n* = 111)	2020 (*n* = 66)	*p*-value
Age (years), median (IQR)	31 (21–49)	37 (23–53)	.12
Sex, *n* (%)			
Male	59 (46.8%)	29 (56%)	
Female	52 (53.2%)	37 (44%)	
Type of surgery, *n* (%)			
Laparoscopic appendectomy	111 (100%)	66 (100%)	
Other	0 (0%)	0 (0%)	
Type of appendicitis, *n* (%)			
Uncomplicated	87 (78.4%)	43 (65.2%)	.05
Complicated	24 (21.6%)	23 (34.8%)	
Perforation	21 (18.9%)	20 (30.3%)	
Abscess	3 (2.7%)	2 (3%)	
Phlegmon	0 (0%)	1 (1.5%)	
Length of symptoms (h), median (IQR)	24 (12–48)	26.5 (14–48)	.82
Delay in surgical treatment (h), median (IQR)	15 (8–20)	14 (7–20)	.96
Length of postoperative stay (h), median (IQR)	24 (19–42)	26 (19–68)	.26
Complications, *n* (%)			
None	102 (91.9%)	63 (95.5%)	.36
Infectious	9 (8.1%)	3 (4.5%)	
Superficial abscess	2 (1.8%)	1 (1.5%)	
Deep abscess	5 (4.5%)	1 (1.5%)	
Colitis	1 (0.9%)	0 (0%)	
Septic thrombophlebitis	1 (0.9%)	1 (1.5%)	

SD, standard deviation; IQR, interquartile range.

All appendicitis were treated laparoscopically with appendectomy. The rates of complicated appendicitis were higher in the COVID cohort (23 patients, 34.8%) as compared with the pre-COVID cohort (24 patients, 21.6%). However, it did not reach any statistical significance (*p* = .05). Perforation was the main presentation of these complicated appendicitis, respectively 30.3% and 18.9% of patients, followed by abscess and phlegmon in both cohorts.

**Figure 3:**
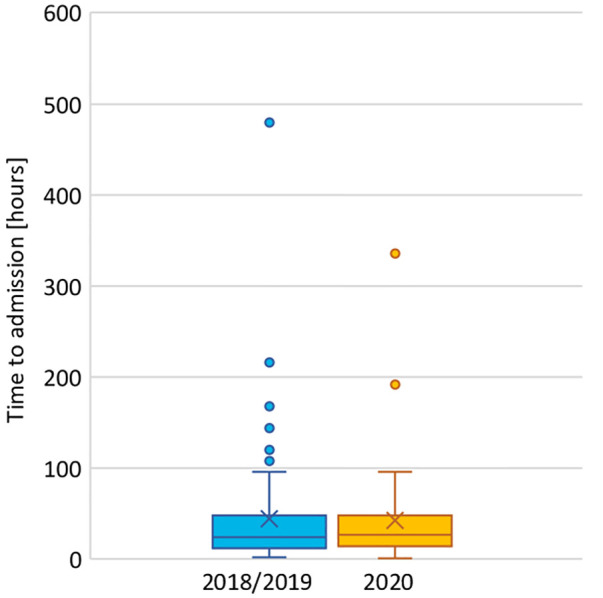
Duration of symptoms before consultation in the emergency room in 2018/2019 and 2020.

Time to emergency department consultation after the first symptoms was 26.5 h (IQR = interquartile range 14–48) in the COVID cohort and 24 h (12–48) in the pre-COVID cohort (*p* = .82). Data are displayed in Figure 3.

**Figure 4:**
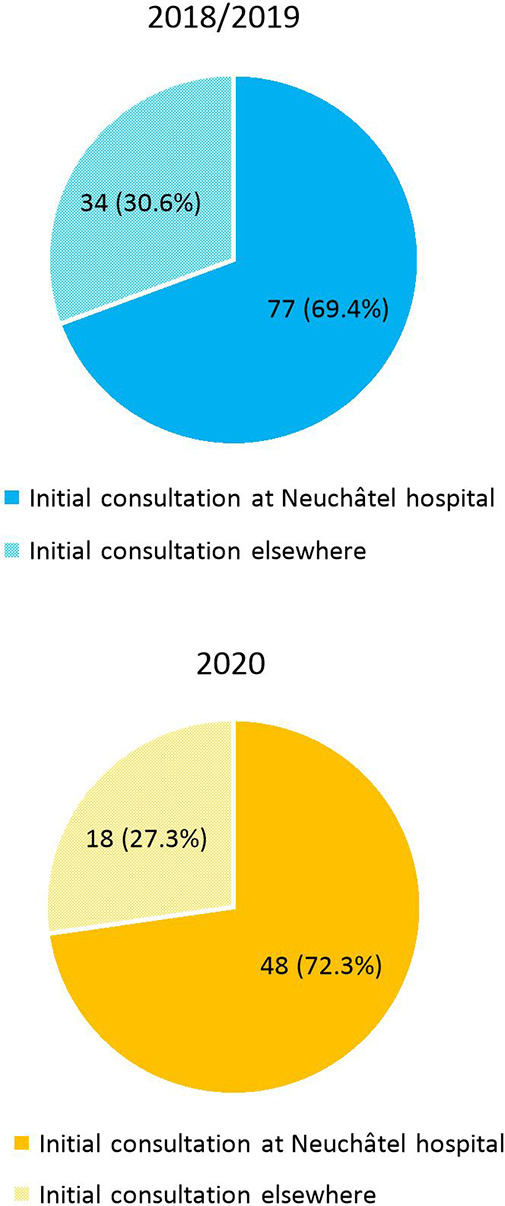
Place of initial consultation: at Neuchâtel hospital or outside the hospital (general practitioner, medical center) in 2018/2019 and 2020.

**Figure 5:**
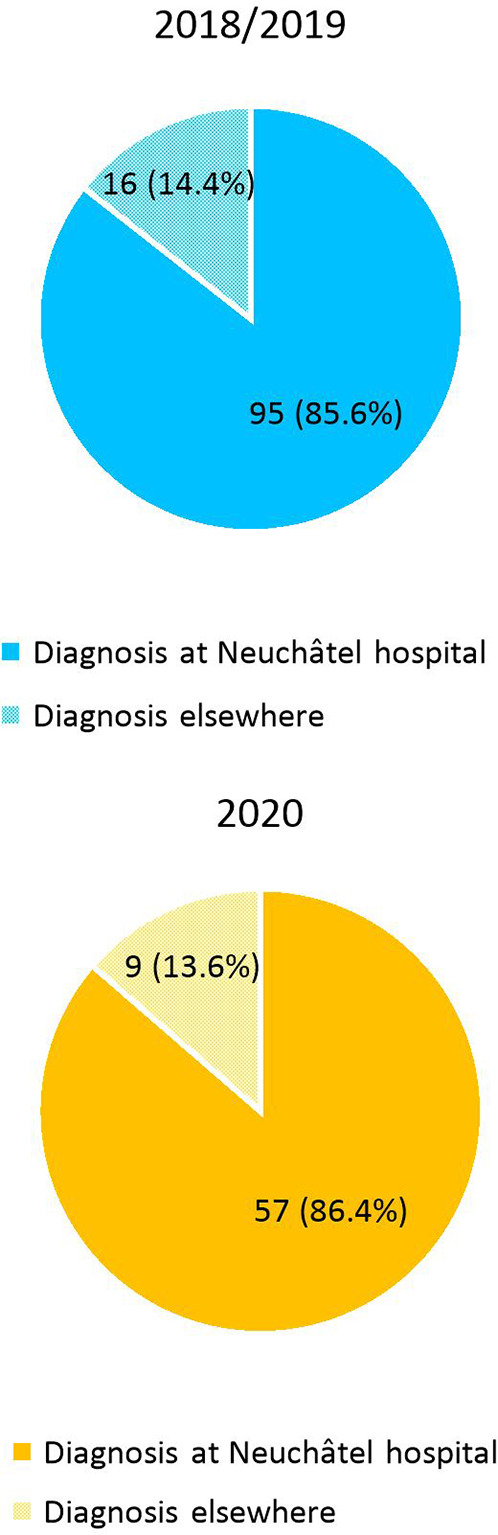
Place of diagnosis on imaging: at Neuchâtel hospital or outside the hospital (general practitioner, medical center) in 2018/2019 and 2020.

Before their admission at the Hospital of Neuchâtel, 18 patients sought medical attention outside the hospital (general practitioner, medical center) in the COVID cohort (27.3%), as compared with 34 patients in the pre-COVID cohort (30.6%, *p* = .63). Among them, 9 and 16 patients were, respectively, diagnosed with acute appendicitis on imaging prior to the emergency room presentation (13.6% of the COVID cohort and 14.4% of the pre-COVID cohort, *p* = .89). These latter results are displayed in Figures 4, 5.

**Figure 6:**
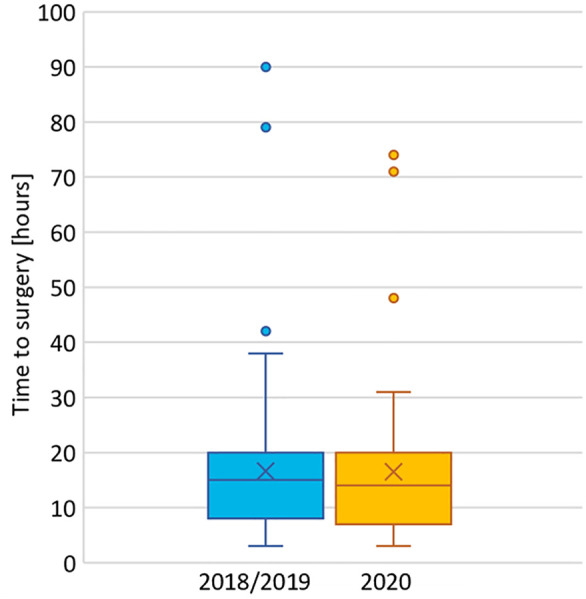
Time from admission at the Hospital of Neuchâtel to surgery in 2018/2019 and 2020.

Median time from admission at the Hospital of Neuchâtel to surgery was 14 h (IQR 7–20) vs. 15 h (8–20) for the COVID cohort and the pre-COVID cohort, respectively (*p* = .96), as seen in Figure 6.

**Figure 7:**
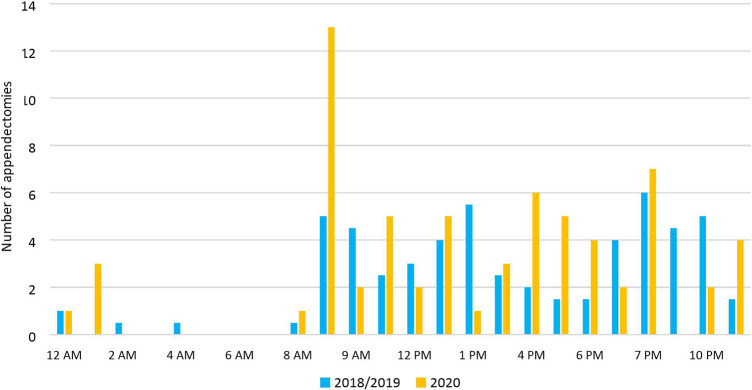
Distribution of appendectomies over a 24-h schedule in 2018/2019 and 2020.

Ten (15.2%) appendectomies were performed outside the usual scheduled time for non-urgent surgery (8 a.m.–10 p.m.) during the pandemic against 17 (15.3%) appendectomies in the pre-COVID cohort, as represented in Figure 7 (*p* = .98).

**Figure 8:**
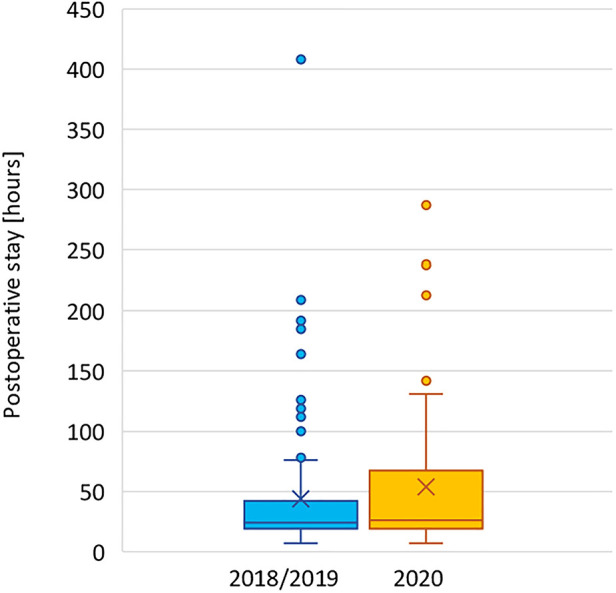
Length of postoperative stay in 2018/2019 and 2020.

Median postoperative hospital stay was 26 h (IQR 19–68) vs. 24 h (19–42) for the two cohorts, respectively (*p* = .26). Data are displayed in Figure 8.

Finally, infectious postoperative complications accounted for 4.5% of cases in the COVID cohort: 1 patient presented a superficial abscess in the COVID cohort, 1 presented a deep abscess, and 1 presented septic thrombophlebitis. The rate of complications in the pre-COVID cohort was 8.1% (*p* = .36): 2 had a superficial abscess, 5 patients had a deep abscess, 1 presented colitis, and 1 septic thrombophlebitis.

As previously discussed, follow-up was achieved either by the surgeon or by Swissnoso upon a phone call, when patients could not meet the surgeon, at 1 month postoperatively. The files of the patients were reviewed up to 4 months to detect any further complications.

No missing data were reported during this study.

## Discussion

Our results showed no statistically significant difference in the number of appendectomies and type of appendicitis during the first two pandemic peaks compared with the previous 2 years. Duration of symptoms and delay to surgery remained unchanged, as well as postoperative complications and length of hospital stay. The operations took place in almost the same 24-h window, despite the greater availability of the emergency operating room during the pandemic.

The absence of a significant decrease in the number of appendectomies in our study might reflect the fact that people would still present to the emergency department for abdominal pain, contrarily to the global tendency observed in the literature. This might be attributed to the fact that the implemented measures in Switzerland were less drastic in terms of social restrictions (no established but recommended lockdown and no curfew). Consequently, people were still ready to present to the emergency department, at least in the canton of Neuchâtel.

Interestingly, our results contrast with the other Swiss studies, as previously described with Passoni and Texeira Farinha (16, 17). Burgard et al. (21) observed an increase in complicated appendicitis and duration of symptoms before presentation during the first pandemic peak. This variation may be attributed to the fact that these teams studied the effects of the COVID-19 pandemic during the first peak, when little was known about the viral disease and people would fear an infection by the virus, which reduced the number of emergency department consultations (22–24). Including the second peak in our study might have reduced the effect of fear of the first peak.

It is to be pointed out that a study made in a tertiary center in Israel also showed that the pandemic did not affect the presentation, clinical course, management, and outcomes of patients with acute appendicitis (25).

Several limitations to this study can be identified. It is designed in a retrospective fashion, and it has a limited sample of patients, the latter affecting the statistical robustness regarding the rates of complicated appendicitis or postoperative complications.

The post-COVID-19 peak from June to September 2020 could have been worth analyzing, as it might have allowed to evaluate the possible delayed impacts of the first peak on the time to presentation and the rates of complicated appendicitis, as reported by Passoni et al. (16).

Including patients diagnosed with acute appendicitis at the emergency department and not only the number of appendectomies would have been valuable. It would have allowed to see how many patients were offered a conservative treatment. However, patients consulting their general practitioner for abdominal pain and not referred to hospital would still constitute a selection bias that would have been difficult to get rid of.

One of the strengths of this study lies in the fact that the Hospital of Neuchâtel is the only institution that offers emergency operating room facility for visceral operations in the canton. The monocentric approach allows to analyze the effects of our operating rooms organization during the pandemic.

In conclusion, the COVID-19 pandemic did not significantly affect the number of appendectomies performed, the clinical course, the surgical management, and the postoperative outcomes.

The reasons might be that people would still present to the emergency department due to less strict social distancing measures. Great availability of emergency operating rooms may also account for the unchanged delay preceding surgical treatment and complication rates.

## Data Availability

The raw data supporting the conclusions of this article will be made available by the authors, without undue reservation.
